# Prognostic Significance of ROR2 Expression in Patients with Urothelial Carcinoma

**DOI:** 10.3390/biomedicines9081054

**Published:** 2021-08-20

**Authors:** Cheng-Fa Yeh, Ti-Chun Chan, Hung-Lung Ke, Tzu-Ju Chen, Li-Ching Wu, Hsiang-Ying Lee, Yu-Ching Wei, Wen-Jeng Wu, Chien-Feng Li, Wei-Ming Li

**Affiliations:** 1 Division of General Internal Medicine, Chi Mei Medical Center, Tainan 710, Taiwan; u802091@gmail.com; 2 Department of Environment Engineering and Science, Chia Nan University of Pharmacy and Science, Tainan 717, Taiwan; 3 Department of Medical Research, Chi Mei Medical Center, Tainan 710, Taiwan; chloe0304@nhri.edu.tw (T.-C.C.); cfli@mail.chimei.org.tw (C.-F.L.); 4 National Institute of Cancer Research, National Health Research Institutes, Tainan 704, Taiwan; 5 Department of Urology, Kaohsiung Medical University Hospital, Kaohsiung 807, Taiwan; hlke@kmu.edu.tw (H.-L.K.); hsiangying@kmu.edu.tw (H.-Y.L.); wejewu@kmu.edu.tw (W.-J.W.); 6 Department of Urology, School of Medicine, College of Medicine, Kaohsiung Medical University, Kaohsiung 807, Taiwan; 7 Department of Urology, Kaohsiung Municipal Ta-Tung Hospital, Kaohsiung 801, Taiwan; 8 Department of Clinical Pathology, Chi Mei Medical Center, Tainan 710, Taiwan; a108n2@mail.chimei.org.tw (T.-J.C.); 540012@mail.chimei.org.tw (L.-C.W.); 9 Department of Pathology, School of Medicine, College of Medicine, Kaohsiung Medical University, Kaohsiung 807, Taiwan; ycwei@kmu.edu.tw; 10 Department of Pathology, Kaohsiung Municipal Ta-Tung Hospital, Kaohsiung 801, Taiwan; 11 Center for Liquid Biopsy and Cohort Research, Kaohsiung Medical University, Kaohsiung 807, Taiwan; 12 Department of Urology, Ministry of Health and Welfare Pingtung Hospital, Pingtung 900, Taiwan

**Keywords:** ROR2, bladder cancer, upper tract urothelial carcinoma, survival, prognosis

## Abstract

We investigated the association of receptor tyrosine kinase-like orphan receptor 2 (ROR2) expression with clinicopathological features and oncologic outcomes in large urothelial carcinoma (UC) of the upper tract (UTUC) and urinary bladder (UBUC) cohorts. Through transcriptomic profiling of a published dataset (GSE31684), ROR2 was discovered to be the most upregulated gene during UC progression, focusing on the JNK cascade (GO:0007254). Initially, the evaluation of *ROR2* mRNA expression in 50 frozen UBUCs showed significantly upregulated levels in high-stage UC. Moreover, high ROR2 immunoexpression significantly correlated with high tumor stage, high tumor grade, lymph node metastasis, and vascular invasion (all *p* < 0.05). In multivariate analysis, after adjusting for standard clinicopathological features, ROR2 expression status was an independent prognosticator of cancer-specific survival and metastasis-free survival in UTUC and UBUC (all *p* < 0.01). In the subgroup analysis, it also significantly predicted bladder tumor recurrence in non-muscle invasive UBUC. Furthermore, the GO enrichment analysis showed that fatty acid, monocarboxylic acid, carboxylic acid metabolic processes, negative regulation of neutrophil migration, and negative regulation of granulocyte and neutrophil chemotaxis were significantly enriched by ROR2 dysregulation. In conclusion, high ROR2 immunoexpression was associated with aggressive pathological characteristics in UC and independently predicted worse prognosis, suggesting it could play roles in clinical risk stratification and therapy decisions.

## 1. Introduction

Urothelial carcinoma (UC) of the upper tract (ureter and renal pelvis) (UT) and urinary bladder (UB) is one of the most frequently occurring malignancies worldwide. UBUC is the 10th most common malignancy globally, with 573,278 estimated new cases in both sexes in 2020, 37% of which (212,536) are predicted to be lethal [1]. Tobacco smoking and occupational exposure to aromatic amines or polycyclic are the main environmental risk factors for UC [2,3,4]. Recent meta-analyses have demonstrated that current and former smokers are at an increased risk of UC. Dose-response analyses indicate that even after long-term smoking cessation, an elevated risk of UC remains [5]. Although family history and heritable gene variants seem to have little impact, genetic studies have demonstrated a potential role of genetic predisposition to urothelial carcinogenesis [6,7].

For UBUC, most non-muscle-invasive bladder cancer (NMIBC) can be treated by the transurethral resection of the bladder tumor (TURBT) and subsequent intravesical instillations [2,3]. Aggressive management, including radical cystectomy, bladder preservation treatment, and perioperative chemotherapy or immunotherapy, are suggested for muscle-invasive bladder cancer (MIBC) or high-risk NMIBC [2,3]. For UTUC, radical nephroureterectomy (RNU) is the standard management of high-risk cancers; kidney-sparing surgery should be considered in all low-risk cases or in select patients with serious renal insufficiency or solitary kidney [4]. Although surgical techniques and therapeutic modalities have significant advancements, the prognosis of patients with UC has not significantlyimproved in the lastthree decades [2,3,4]. Therefore, it is vital to discover new prognostic biomarkers and elucidate the UC progression mechanism.

Emerging evidence shows that c-Jun N-terminal kinase (JNK) promotes tumor progression and is involved in various cancers [8,9]. JNK-associated signaling pathways also modulate metabolic reprogramming, cancer stem cells, tumor proliferation, and migration [8,9,10,11]. Focusing on the genes belonging to the JNK cascade (GO:0007254) and associated cancer progression, data mining was performed on a public dataset of bladder cancer. These genes are listed in Tables S1–S5. Some of them, such as *MLK3*, *TAK1*, *ASK1*, *MKK4*, *MKK7*, *JNK1*, and *JNK2*, have been demonstrated to regulate cell proliferation, cell survival, cell differentiation, and cell renewal [8,9].We found that receptor tyrosine kinase-like orphan receptor 2 (ROR2) was the most upregulated gene during bladder cancer progression, from NMIBC to MIBC.

ROR2, a novel Wnt receptor, is normally expressed at high levels during development and morphogenesis and plays a key role in chondrogenesis, plate development, chondrogenesis, and growth plate development [12]. It is a highly pleiotropic receptor with a complex role in human carcinogenesis. The upregulation of ROR2 has been established in a multitude of tumor types, such as osteosarcoma, prostate cancer, and renal cell carcinoma [13,14,15]. In contrast, ROR2 acts to suppress carcinogenesis in colon cancer and hepatocellular carcinoma [16,17]. However, the roles of ROR2 in UC have not been well studied. We aimed to elucidate the association of ROR2 with clinicopathological features and the prognostic impact of UC in our cohort.

## 2. Materials and Methods

### 2.1. Public Data

The UBUC patient data (GSE31684) retrieved from the Gene Expression Omnibus (GEO) data repository was a transcriptomic public microarray dataset measured on the Affymetrix Human Genome HG-U133 Plus 2.0 array. We processed the data as previously reported [18,19]. Raw files were imported into the Nexus Expression 3 software (BioDiscovery, El Segundo, CA, USA) without pre-selection to computerize the expression level. To identify the cancer progression-associated genes, we calculated the differentially expressed genes (DEGs) focusing on the JNK cascade (GO:0007254) between MIBC and NMIBC. We selected the top six DEGs (*p* < 0.01 and |log2 ratio| > 0.5) for further research.

### 2.2. Patient Data and Tissues

After Institutional Review Board approval (IRB10501-005) on 4 February 2016, we identified 635 UC patients, and 295 UBUC and 340 UTUC patients were treated with surgery with curative intent between 1998 and 2004. None received neoadjuvant chemotherapy or radiotherapy before surgery. The performance and extent of lymphadenectomy were based on the surgeon’s discretion. All surgical specimens were processed per the standard pathological procedures. The pathological stage was reassigned on the basis of the 2010 AJCC/UICC classification, and tumor grade was evaluated using the 2004WHO/ISUP consensus criteria. Comprehensive clinical, pathological, and follow-up data elements were retrospectively reviewed for each patient.

### 2.3. Quantitative Real-Time PCR

Using the Total RNA Purification Kit (GeneMark, Atlanta, GA, USA), total RNA extraction was performed, and cDNA was obtained using the Maxima First Strand cDNA Synthesis Kit (Thermo Fisher Scientific, Waltham, MA, USA) according to the manufacturer’s instructions. Quantitative real-time PCR (qRT-PCR) was performed on a StepOne Plus System (Applied Biosystems, Waltham, MA, USA) as previously described [18,19]. We calculated the fold expression of ROR2 relative to the adjacent non-tumor urothelium using the ΔΔCT method after normalization to the endogenous control gene, POLR2A.The following TaqMan™ Gene Expression assay probes (Thermo Fisher Scientific, Waltham, MA, USA) were used: ROR2 (assay ID:Hs00896176_m1, Catalog No.:4331182) and POLR2A (assay ID:Hs01108291_m1, Catalog No.:4331182).

### 2.4. Immunohistochemistry

We followed the standard immunohistochemistry (IHC) protocols as described previously [20,21]. Four-micrometer-thin sections were cut from formalin-fixed, paraffin-embedded tissue blocks. The samples were then incubated with anti-ROR2 primary antibody (1:100, ab-218105, Abcam).We detected IHC reactions using the DakoREALEnVision™ detection system. Slides were analyzed by a manual reading of two dedicated pathologists who were blinded to the clinicopathological features and patient outcomes. They appraised the intensity (0:negative; 1+:weak; 2+:moderate; 3+: strong) and percentage (0 to 100%) of positive immunostaining UC cells to generate the H-score, using the following equation: H-score = 1 × (% of weakly stained cells) +2 × (% of moderately stained cells)+ 3× (% of strongly stained cells). An H-score between 0 and 300 was obtained, wherein 300 equals 100% of the tumor cell staining corresponding to strongly positive (3+) staining. We divided immunoreactivity into low and high expression levels using the median H-score.

### 2.5. Gene Ontology (GO) Enrichment Analysis

We used the gene expression information from the TCGA_BLCA database of the cBioPortal platform (https://www.cbioportal.org/) accessed on 1 December 2020 and screened for ROR2 positive and negative co-expression genes. Spearman correlation coefficient was calculated. The top 500 co-expressed genes were selected for GO enrichment analysis to understand the function of ROR2 co-expression genes on the gene-ontology website (https://geneontology.org/) accessed on 1 December 2020, which was powered by the PANTHER classification system.

### 2.6. Statistical Analysis

Associations between ROR2 expression and patient or UC characteristics were determined using a chi-square test. Oncologic outcomes, including disease-specific survival (DSS), metastasis-free survival (MFS), and bladder recurrence-free survival (BRFS), were measured from the date of surgery to the date of events that occurred. Survivors at the end of the study were censored at the date of the last follow-up. These outcomes were compared using Kaplan–Meier methods stratified by the ROR2 expression status (high vs. low) using the log-rank statistic. We used multivariate Cox proportional hazard regression models to identify the independent predictors of oncologic outcomes (DSS, MFS, and BRFS). All statistical analyses were conducted using SPSS (version 17.0; IBM, Armonk, NY, USA). All tests were 2-sided, and *p* values < 0.05 were considered statistically significant.

## 3. Results

### 3.1. Data Mining of Significantly Altered Genes Belong to the JNK Cascade in UC Progression

**Figure 1:**
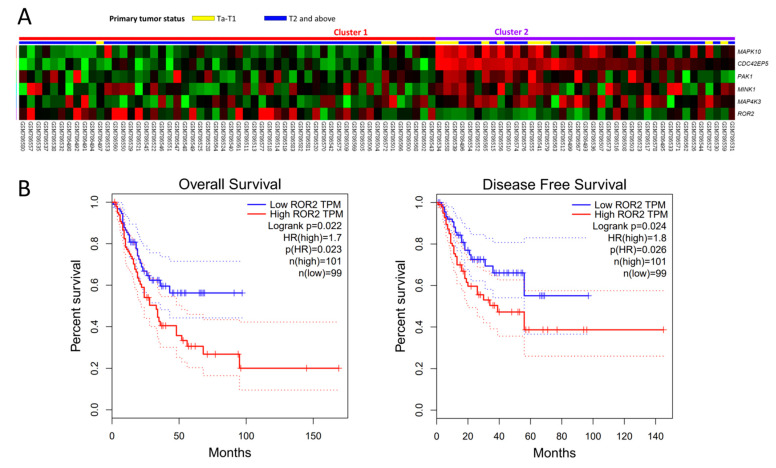
Transcriptomic profiling and survival analysis. (**A**) Expression profiles of genes associated with the progression of UBUC (T2-4 vs. Ta-1) focusing on JNK cascade from a published transcriptome (GSE31684) in Gene Expression Omnibus. ROR2 is the most significantly upregulated gene. (**B**) Using GEPIA databases, high *ROR2* expression was remarkably related to worse overall survival (*p* = 0.022) and disease-free survival (*p* = 0.024) in UBUC.

**Figure 2:**
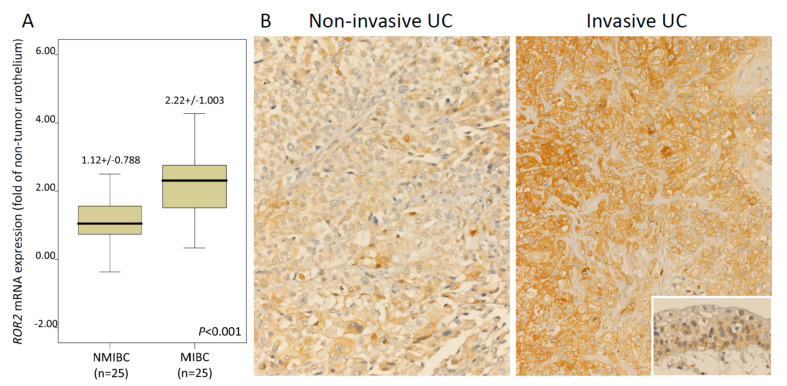
Expression of ROR2 mRNA and protein in urothelial carcinoma specimens. (**A**) *ROR2* mRNA level was significantly increased in MIBC (pT2-T4) using qRT-PCR. (**B**) Invasive UC showed high ROR2 expression using immunohistochemistry (normal urothelium in the inset) (magnification × 2000).

Through transcriptomic profiling of a GEO dataset (GSE31684), we identified the top six significantly dysregulated genes belonging to the JNK cascade associated with muscle invasion in UBUC (Table 1 and Figure 1A). The prognostic potentials of these genes were assessed using GEPIA databases. Only high *ROR2* expression is related to worse overall survival (*p* = 0.022) and disease-free survival (*p* = 0.024) (Figure 1B). The expression status of the other five genes is not associated with patient outcomes (Figure S1). Therefore, we selected ROR2 for further study. Initially, the *ROR2* transcript was evaluated in 50 snap-frozen UBUC tissues. *ROR2* mRNA showed significantly high expression in MIBC (*p* < 0.001), signifying its function in UC aggressiveness (Figure 2A). These findings prompted us to further study the correlations between the ROR2 protein level and clinicopathological features and its prognostic roles in our large, well-characterized UTUC and UBUC cohorts.

### 3.2. Descriptive Characteristics and Association with ROR2 Status

Table 2 shows the association between ROR2 expression and clinicopathological features. Representative examples of ROR2 staining are shown in Figure 2B.

In the UTUC group, we included 340 patients, with 53.5% of male patients, and the mean age at the time of surgery was 65.58 ± 9.89 years. Gender distribution, age, tumor location, multifocality, perineural invasion, and mitotic rate were similar between the high and low expression groups. High ROR2 expression was more often associated with advanced pathologic T stage (*p* < 0.001), nodal metastasis (*p* < 0.001), high histological grade (*p* = 0.008) and vascular invasion (*p* < 0.001).

We included 295 patients in the UBUC cohort. The median age was 67 years, and most patients were men (73.2%). There was no difference in sex, age, lymph node status, perineural invasion, and mitotic rate between patients with high and low ROR2expression. Tumors with high ROR2 expression by IHC showed a high primary tumor stage (*p* < 0.001), high histological grade (*p* < 0.001), and vascular invasion (*p* = 0.045).

### 3.3. Prognostic Significance of ROR2 Immunoexpression

**Figure 3:**
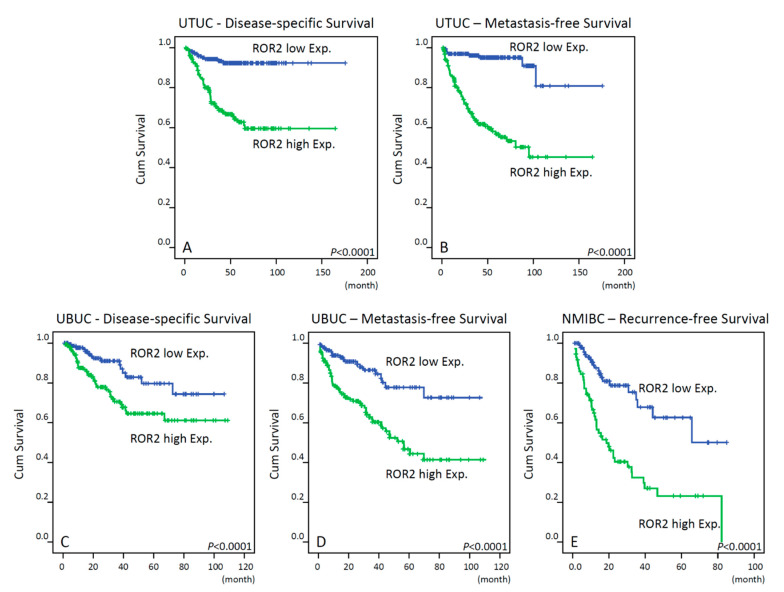
Kaplan–Meier survival analysis shows that ROR2 overexpression has significant prognostic impacts in disease-specific survival, metastasis-free survival, and bladder recurrence-free survival of patients with UTUC (**A**,**B**, respectively) and UBUC (**C**,**D**, respectively) and bladder recurrence-free survival of patients with NMIBC (**E**).

In the UTUC cohort, the median follow-up time was 38.2 months (mean 44.7± 31.9 ms). During this period, 61 patients (25.2%) experienced cancer-related death, and 70 patients (34.5%) had tumor metastasis. Kaplan–Meier analysis revealed that patients with high ROR2 expression had a lower probability of DSS (*p* < 0.0001; Figure 3A) and MFS (*p* < 0.0001; Figure 3B) compared to those with low ROR2 expression. In the multivariable analyses (Table 3), adjusted for the effects of standard clinicopathological features, demonstrated that ROR2 expression was significantly associated with the probability of disease-specific mortality (HR:3.302; 95% CI = 1.621–6.727; *p* = 0.001) and metastasis (HR:6.691; 95% CI = 3.181–14.075; *p* < 0.001).

In the UBUC cohort, the median follow-up time was 23.4 months (mean 30.8 ± 25.6 ms). Over a median follow-up of 23.4 months, 76 patients (26.7%) experienced cancer metastasis, and 52 (18.2%) died subsequently from UBUC. As shown in Figure 3, high ROR2 expression was significantly associated with worse MFS (*p* < 0.0001; Figure 3C) and DSS (*p* < 0.0001; Figure 3D) in Kaplan–Meier analysis. Predictors of oncologic outcomes identified using Cox regression analyses are summarized in Table 4 for DSS and MFS. Inthe multivariate analysis, after adjusting for primary tumor stage, tumor grade, perineural invasion, vascular invasion, lymph node status, and mitotic rate for MFS and CSS, we found that ROR2 expression status remained an independent predictor of CSS (HR:2.166; 95% CI = 1.178–3.983; *p* = 0.013) and MFS (HR: 2.786; 95% CI = 1.646–4.714; *p* < 0.001). The other independent predictors of DSS and MFS are listed in Table 4.

We performed a subgroup analysis in 172 patients with NMIBC. Of these patients, 65 (37.8%) had urinary bladder tumor recurrence. In Kaplan–Meier analysis, ROR2 high expression was associated with a high urinary bladder tumor recurrence rate (*p* < 0.0001; Figure 3E). In the multivariate analysis, we found that ROR2 expression status (HR: 3.033; 95% CI = 1.758–5.233, *p* < 0.001) and primary tumor stage and grade were independent predictors for urinary bladder tumor recurrence in patients with NMIBC (Table 5).

### 3.4. GO Enrichment Analysis of ROR2 Co-Expressed Genes in UC

ROR2 co-expressed genes were calculated by analyzing their mRNA expression using the cBioPortal online tool for TCGA-BLCA with Spearman’s correlation. The top 500 positive and 500 negative co-expressed genes are listed in Tables S2 and S3. GO enrichment analysis was performed using the gene ontology resource online tool (Tables S4 and S5). For the biological process function, the genes were mainly enriched in fatty acid, monocarboxylic acid, and carboxylic acid metabolic processes, negative regulation of neutrophil migration, and negative regulation of granulocyte and neutrophil chemotaxis. Taken together, these results suggest that ROR2 may play crucial biological roles in UC metabolic reprogramming and immune microenvironment regulation.

## 4. Discussion

UC is one of the most frequently diagnosed, heterogeneous, and harmful cancers worldwide. Some tissue-based biomarkers have also been assessed for their prognostic value in UC, but little is utilized routinely in clinical practice to guide individual treatment decisions. In this study, we assessed the association of ROR2 expression with the pathological features of UTUC and UBIC and its value in prognosticating DSS, MFS, and BRFS BCR in a large UC cohort. We found that ROR2 high expression was associated with established features of biologically and clinically aggressive UC, including high pathologic stage, high histological grade, nodal metastasis, vascular invasion, perineural invasion, and high mitotic activity which are often linked to poor clinical outcomes. ROR2 expression in UC may define a subset of high-risk tumors, which may require additional treatment.

The association between ROR2 expression and patient outcomes has been studied in some malignancies. Edris et al. determined whether ROR2 expression using IHC was associated with patient outcomes in sarcomas [22]. They found that ROR2 immunoexpression predicts poor overall survival and DSS in patients with gastrointestinal stromal tumor and leiomyosarcoma. However, in a multivariate analysis, ROR2 was not independent of other clinicopathological characteristics [22]. Gou et al. found that ROR2 gene mRNA expression was significantly increased in breast cancer tissues compared with corresponding non-tumor tissues [23]. High ROR2 gene mRNA expression had a large tumor size and reducedthe disease-free survival rate. Henry et al. showed ROR2 protein expression in most breast cancer patients (87%) but not in normal breast tissue [24]. Its high expression confers a poorer DSS, especially in triple-negative breast cancer patients. In lung cancer, Lu et al. showed that ROR2 mRNA expression and protein were significantly increased in lung cancer [25]. Patients with high ROR2 expression are significantly associated with advanced cancer stage, lymph node metastasis, and a poorer survival rate after the operation. In this study, we found that ROR2 expression was associated with recurrence, metastases spread, and cancer-specific mortality in univariate analysis; moreover, after adjusting for standard prognostic factors in multivariate analysis, the ROR2 expression status remained associated with poor outcomes, implying its prognostic value.

In UBUC, for effectively treating NMIBC, the identification of high-risk patients who may progress to MIBC is a critical challenge [2,3]. We observed that ROR2 immunoexpression may be a biomarker for identifying these patients, as significantly high ROR2 expression was observed in muscle-invasive and high-grade UBUC. Patients with high ROR2 expression have three-times greater bladder tumor recurrence rate than those with low ROR2 expression, suggesting its prognostic role in NMIBC. The early identification of the patients with metastatic potential is another important issue. Our results demonstrated that UBUC patients with high ROR2 expression and aggressive pathological features were more likely to develop lymph node and distant metastasis. Patients with high ROR2-expressing UBUCs may receive more aggressive management, such as early radical surgery and perioperative systemic chemotherapy or immunotherapy.

In UTUC, kidney-sparing surgery is suggested for low-risk cancers due to the low surgical complication rate and comparable survival rate to RNU [4]. Along with our findings, high ROR2-expressing UTUC is associated with aggressive cancer characteristics and worse outcomes. Therefore, RNU should be considered in low-risk UTUC patients but with high ROR2 immunoexpression. Lymphadenectomy and adjuvant/neoadjuvant chemotherapy improve local recurrence and survival rate in patients with advanced UTUC [4,26]. We found that high ROR2 expression tumors were significantly associated with muscle-invasive or lymph node metastatic UTUC and a high cancer death rate. These high-risk UTUC patients may receive an aggressive treatment protocol, RNU with lymphadenectomy, and perioperative systemic chemotherapy.

Although the detailed molecular mechanism of ROR2 in UC progression is not well understood, some regulatory pathways have been discovered in other malignancies. Many studies demonstrate that ROR2 activates WNT signaling responses by binding the WNT5A ligand [12,27]. Furthermore, ROR2 facilitates breast cancer progression by regulating the expression of PI3K/AKT and apoptotic signaling genes [4]. In detail, ROR2 overexpression promotes PI3K activation and AKT phosphorylation, followed by the downregulation of p21 and upregulation of cyclin D1 and PDK1, resulting in increased proliferation and survival of breast cancer cells [23]. ROR2 also regulates the epithelial-mesenchymal transition phenotype of breast cancer cells via the activation of the MAPK/p38 signaling pathway [28]. The overexpression of ROR2 significantly upregulated snail, N-cadherin, and vimentin but reduced E-cadherin expression [28].

The unique and critical role of the ROR2 makes it an ideal target for therapeutic intervention [27]. The ongoing study of ROR2 targeting therapy deals with adoptive immunity. Peng et al. designed a rabbit monoclonal antibody XBR2-401, which has valuable preclinical results owing to the similarity of the extracellular domains of rabbit and human ROR2. Afterward, they developed the monoclonal antibody into a chimeric antigen receptor (CAR) T cell format and designed the T cell-engaging ROR2 × CD3 bispecific antibodies (biAbs), which had a high specificity towards ROR2 [29,30]. Advanced investigations regarding ROR2-targeting antibody-drug conjugates are already underway. One potential candidate is BA3021, a conditionally active biological (CAB) ROR2-targeted antibody-drug conjugate (CAB-ROR2-ADC). It is now being assessed in a multicenter, open-label, phase 1/2 study in patients with advanced solid tumors (NCT03504488). Two additional active clinical trials are ongoing to investigate ROR2-specific CAR T cells in ROR2-positive renal cell carcinomas (NCT03393936) and ROR2 expressing solid malignancies (NCT03960060).

JNKs are important genes in the JNK cascade. There are three differently spliced genes of JNK, namely, *JNK1(MAPK8)*, *JNK2 (MAPK9)*, and *JNK3(MAPK10)* [9]. The difference between ROR2 and JNKs is that ROR2 is a receptor protein tyrosine kinase and type I transmembrane protein, while JNKs are mitogen-activated protein kinases and intracellular proteins [9,12]. In our analysis, JNK3(MAPK10)there was a negative association with UC invasiveness (Table 1). Using GEPIA, we evaluated the prognostic roles of JNK1, JNK2, and JNK3 in UC. The results showed JNKs genes expression status was not associated with overall survival (Figure S2). JNK could have both oncogenic and tumor suppressing roles depending on the cancer type and stage [9,31]. Currently, most studies use pan-JNK inhibitorsin vitro, which lack specificity and indiscriminately inhibit phosphorylation of all JNK substrates [8,32]. Due to lack of efficacy and unpredicted side effects, most clinical trials of JNK inhibitors were unsuccessful [8,32]. Therefore, the development of selective JNK inhibitors are wanted to be a valuable cancer therapeutic strategy.

There are some limitations in our study, including those inherent to any retrospective study. Another limitation is that IHC results may be inconsistent due to the variability in the methodology and scoring protocols. We used a semiquantitative method, combining staining density and percentage, to evaluate immune expression activity by two independent pathologists to maximize the reliability of the results. IHC is also an inexpensive test and is available in most laboratories. Moreover, we did not elucidate the clear molecular mechanism of ROR2 and the therapeutic efficacy of ROR2 targeting therapy in UC.Further researches are needed to focus on these issues.Finally, although our UC cohort number was quite large, further validation may be necessary to confirm our results in a multi-institutional prospective design.

## 5. Conclusions

High ROR2 expression is associated with aggressive clinicopathological features in patients with UTUC and UBUC treated with curative intent. This is the first study to demonstrate that ROR2 expression is an independent prognosticator of DSS, MFS, and BRFS in UC after adjustment with standard pathological characteristics. The assessment of ROR2 expression by standard immunostaining appears to be valuable in clinical practice for patient stratification. ROR’s biological role in UC pathogenesis remains to be elucidated and may be helpful as a therapeutic target.

## Figures and Tables

**Table 1:** The top six significantly dysregulated genes belonging to the JNK cascade are associated with muscle invasion in UBUC (GSE31684).

Probe	Comparing MIBC vs. NMIBC		Gene Symbol	Gene Title	Biological Process
	Log Ratio	*p*-Value			
204813_at	–0.9123	0.0024	*MAPK10*	mitogen-activated protein kinase 10	JNK cascade, protein amino acid phosphorylation, signal transduction
205578_at	0.6393	<0.0001	*ROR2*	receptor tyrosine kinase-like orphan receptor 2	JNK cascade, Wnt receptor signaling pathway; calcium modulating pathway, cartilage condensation, cell differentiation, embryonic genitalia morphogenesis, multicellular organismal development, protein amino acid phosphorylation, signal transduction, skeletal development, somitogenesis
214246_x_at	–0.5616	0.0083	*MINK1*	misshapen-like kinase 1 (zebrafish)	JNK cascade, multicellular organismal development, negative thymic T cell selection, protein amino acid phosphorylation, protein kinase cascade, response to stress
218311_at	–0.5919	0.0072	*MAP4K3*	mitogen-activated protein kinase kinase kinase kinase 3	JNK cascade, protein amino acid phosphorylation, protein kinase cascade, response to stress
227850_x_at	–1.6026	<0.0001	*CDC42EP5*	CDC42 effector protein (Rho GTPase binding) 5	JNK cascade, Rho protein signal transduction, positive regulation of actin filament polymerization, positive regulation of pseudopodium formation, regulation of cell shape
230100_x_at	–0.6927	0.0011	*PAK1*	p21/Cdc42/Rac1-activated kinase 1 (STE20 homolog; yeast)	ER-nuclear signaling pathway, JNK cascade, apoptosis, cytoskeleton organization and biogenesis, dendrite development, protein amino acid phosphorylation

**Table 2:** Correlations between ROR2 expression and other important clinicopathological parameters in urothelial carcinomas.

Parameter	Category	Upper Urinary Tract Urothelial Carcinoma				Urinary Bladder Urothelial Carcinoma			
		Case No.	ROR2 Expression		*p*-Value	Case No.	ROR2 Expression		*p*-Value
			Low	High			Low	High	
Gender	Male	158	71	87	0.082	216	110	106	0.534
	Female	182	99	83		79	37	42	
Age (years)	<65	138	69	69	1.000	121	59	62	0.759
	≥65	202	101	101		174	88	86	
Tumor location	Renal pelvis	141	63	78	0.232	-	-	-	-
	Ureter	150	82	68		-	-	-	-
	Renal pelvis & ureter	49	25	24		-	-	-	-
Multifocality	Single	278	137	141	0.574	-	-	-	-
	Multifocal	62	33	29		-	-	-	-
Primary tumor (T)	Ta	89	57	32	<0.001 *	84	56	28	<0.001 *
	T1	92	54	38		88	43	45	
	T2–T4	159	59	100		123	48	75	
Nodal metastasis	Negative (N0)	312	165	147	<0.001 *	266	136	130	0.177
	Positive (N1–N2)	28	5	23		29	11	18	
Histological grade	Low grade	56	37	19	0.008 *	56	40	16	<0.001 *
	High grade	284	133	151		239	107	132	
Vascular invasion	Absent	234	138	96	<0.001 *	246	129	117	0.045 *
	Present	106	32	74		49	18	31	
Perineural invasion	Absent	321	162	159	0.479	275	139	136	0.362
	Present	19	8	11		20	8	12	
Mitotic rate (per 10 high power fields)	<10	173	92	81	0.233	139	71	68	0.686
	≥10	167	78	89		156	76	80	

* Statistically significant.

**Table 3:** Univariate log-rank and multivariate analyses for disease-specific and metastasis-free survival in upper urinary tract urothelial carcinoma.

Parameter	Category	Case No.	Disease-Specific Survival					Metastasis-Free Survival				
			Univariate Analysis		Multivariate Analysis			Univariate Analysis		Multivariate Analysis		
			No. of Event	*p*-Value	R.R.	95% CI	*p*-Value	No. of Event	*p*-Value	R.R.	95% CI	*p*-Value
Gender	Male	158	28	0.8286	-	-	-	32	0.7904	-	-	-
	Female	182	33		-	-	-	38		-	-	-
Age (years)	<65	138	26	0.9943	-	-	-	30	0.8470	-	-	-
	≥65	202	35		-	-	-	40		-	-	-
Tumor side	Right	177	34	0.7366	-	-	-	38	0.3074	-	-	-
	Left	154	26		-	-	-	32		-	-	-
	Bilateral	9	1		-	-	-	0		-	-	-
Tumor location	Renal pelvis	141	24	0.0079 *	1	-	0.934	31	0.0659	-	-	-
	Ureter	150	22		0.864	0.467–1.601		25		-	-	-
	Renal pelvis& ureter	49	15		1.430	0.392–5.215		14		-	-	-
Multifocality	Single	273	48	0.0026 *	1	-	0.005 *	52	0.0127 *	1	-	<0.001 *
	Multifocal	62	18		3.026	1.400–6.539		18		2.897	1.657–5.065	
Primary tumor (T)	Ta	89	2	<0.0001 *	1	-	0.035 *	4	<0.0001 *	1	-	0.037 *
	T1	92	9		4.810	1.009–22.935		15		4.390	1.403–13.739	
	T2–T4	159	50		5.640	1.223–26.013		51		2.990	0.920–9.723	
Nodal metastasis	Negative (N0)	312	42	<0.0001 *	1	-	<0.001 *	55	<0.0001 *	1	-	0.010 *
	Positive (N1-N2)	28	19		4.062	2.187–7.535		15		2.262	1.217–4.205	
Histological grade	Low grade	56	4	0.0215 *	1	-	0.052	3	0.0027 *	1	-	0.265
	High grade	284	57		2.701	0.933–7.348		67		1.592	0.703–3.606	
Vascular invasion	Absent	234	24	<0.0001 *	1	-	0.309	26	<0.0001 *	1	-	0.016 *
	Present	106	37		1.371	0.746–2.518		44		2.186	1.156–4.132	
Perineural invasion	Absent	321	50	<0.0001 *	1	-	<0.001 *	61	<0.0001 *	1	-	0.001 *
	Present	19	11		4.753	2.274–9.931		9		3.634	1.706–7.740	
Mitotic rate (per 10 high power fields)	<10	173	27	0.167	-	-		30	0.0823	-	-	
	≥10	167	34		-	-		40		-	-	
ROR2 expression	Low	170	11	<0.0001 *	1	-	0.001 *	9	<0.0001 *	1	-	<0.001 *
	High	170	45		3.302	1.621–6.727		61		6.691	3.181–14.075	

* Statistically significant.

**Table 4:** Univariate log-rank and multivariate analyses for disease-specific and metastasis-free survival in urinary bladder urothelial carcinoma.

Parameter	Category	Case No.	Disease-Specific Survival					Metastasis-Free Survival				
			Univariate Analysis		Multivariate Analysis			Univariate Analysis		Multivariate Analysis		
			No. of Event	*p*-Value	R.R.	95% CI	*p*-Value	No. of Event	*p*-Value	R.R.	95% CI	*p*-Value
Gender	Male	216	41	0.4446	-	-	-	60	0.2720	-	-	-
	Female	79	11		-	-	-	16		-	-	-
Age (years)	<65	121	17	0.1136	-	-	-	31	0.6875	-	-	-
	≥65	174	35		-	-	-	45		-	-	-
Primary tumor (T)	Ta	84	1	<0.0001 *	1	-	<0.001 *	4	<0.0001 *	1	-	0.001 *
	T1	88	9		5.708	0.612–53.214		23		4.603	1.317–16.085	
	T2–T4	123	42		24.526	2.818–213.432		49		7.448	2.182–25.426	
Nodal metastasis	Negative (N0)	266	41	0.0002 *	1	-	0.194	61	<0.0001 *	1	-	0.167
	Positive (N1–N2)	29	11		1.602	0.787–3.263		15		2.108	1.132–3.925	
Histological grade	Low grade	56	2	0.0013 *	1	-	0.969	5	0.0007 *	1	-	0.614
	High grade	239	50		0.970	0.211–4.462		71		1.031	0.365–2.910	
Vascular invasion	Absent	246	37	0.0024 *	1	-	0.136	54	0.0001 *	1	-	0.928
	Present	49	15		0.581	0.285–1.187		22		0.935	0.507–1.724	
Perineural invasion	Absent	275	44	0.0001 *	1	-	0.067	66	0.0007 *	1	-	0.326
	Present	20	8		2.226	0.946–5.240		10		1.472	0.691–3.136	
Mitotic rate (per 10 high power fields)	<10	139	12	<0.0001 *	1	-	0.013 *	23	<0.0001 *	1	-	0.012 *
	≥10	156	40		2.381	1.198–4.732		53		1.967	1.162–3.329	
ROR2 expression	Low	147	9	<0.0001 *	1	-	0.013 *	19	<0.0001 *	1	-	<0.001 *
	High	148	43		2.166	1.178–3.983		57		2.786	1.646–4.714	

* Statistically significant.

**Table 5:** Univariate log-rank and multivariate analyses for bladder recurrence-free survival in NMIBC post TURBT.

Parameter	Category	Case No.	Bladder Recurrence-Free Survival				
			Univariate Analysis		Multivariate Analysis		
			No. of Event	*p*-Value	R.R.	95% CI	*p*-Value
Gender	Male	125	46	0.3370	-	-	-
	Female	47	19		-	-	-
Age (years)	<65	70	30	0.3857	-	-	-
	≥65	102	35		-	-	-
Primary tumor (T)	Ta	84	27	0.0193 *	1	-	0.765
	T1	88	38		1.098	0.597–2.019	
Histological grade	Low grade	54	15	0.0101 *	1	-	0.261
	High grade	118	50		1.513	0.735–3.115	
Vascular invasion	Absent	171	65	0.6639	-	-	-
	Present	1	0		-	-	-
Perineural invasion	Absent	169	64	0.4725	-	-	-
	Present	3	1		-	-	-
Mitotic rate (per 10 high power fields)	<10	94	35	0.1853	-	-	-
	≥10	78	30		-	-	-
ROR2 expression	Low	99	20	<0.0001 *	1	-	<0.001 *
	High	73	45		3.033	1.758–5.233	

* Statistically significant.

## Data Availability

All data generated or analyzed during this study are included in this published article and its Supplementary Documentation File.

## Supplementary Materials

The following are available online at https://www.mdpi.com/article/10.3390/biomedicines9081054/s1. Table S1: The genes belonging to the JNK cascade (GO:0007254). Table S2: The top 500 most significant differentially expressed genes that are positively correlated with *ROR2* expression in bladder cancer. Table S3: The top 500 most significant differentially expressed genes that are negatively correlated with *ROR2* expression in bladder cancer. Table S4: GO enrichments of positively associated genes. Table S5: GO enrichments of negatively associated genes. Figure S1: Kaplan–Meier analysis of overall survival according to *MAPK10*, *MINK1*, *MAP4K3*, *CDC42EP5*, and *PAK1* expression in UC patients. Figure S2: Kaplan–Meier analysis of overall survival according to *JNKs* expression in UC patients.
