# Supplementary figures and images for: Prognostic Significance of ROR2 Expression in Patients with Urothelial Carcinoma
Source: Biomedicines. 2021 Aug 20;9(8):1054. doi: 10.3390/biomedicines9081054 (PMC8392262; doi:10.3390/biomedicines9081054)

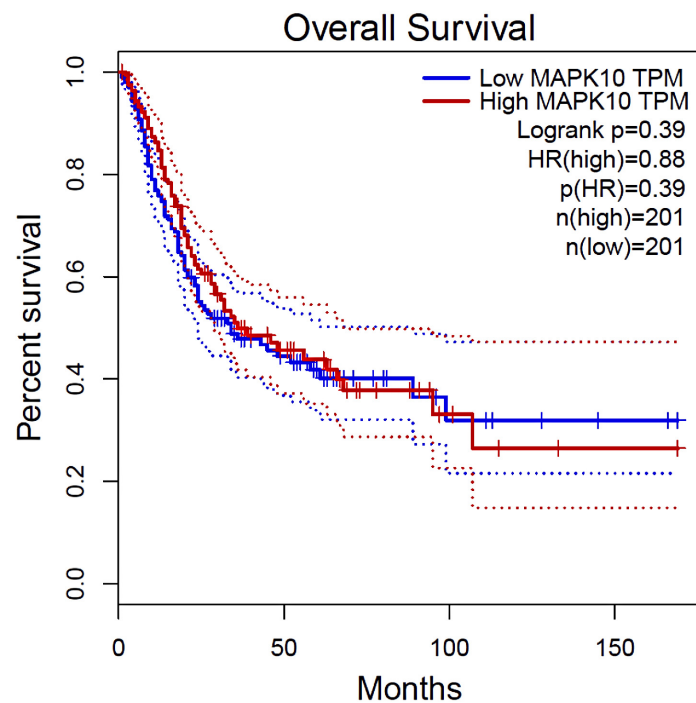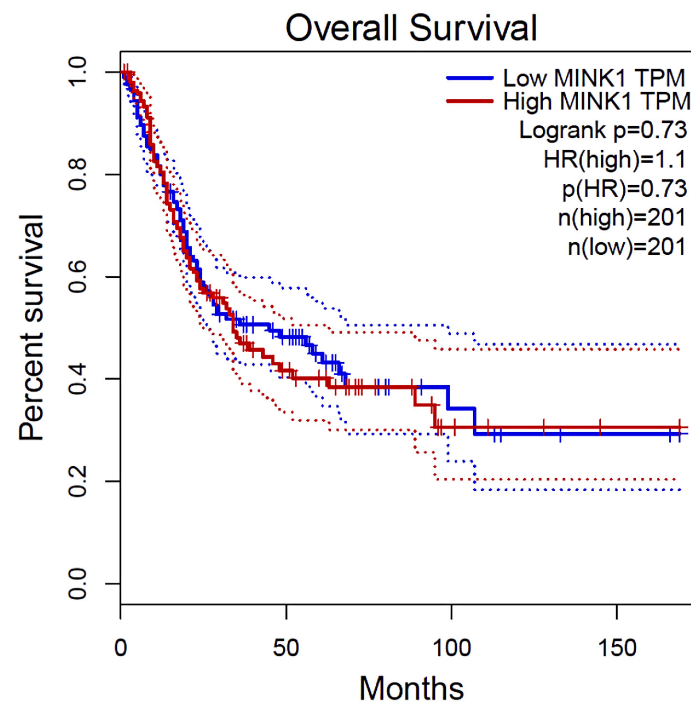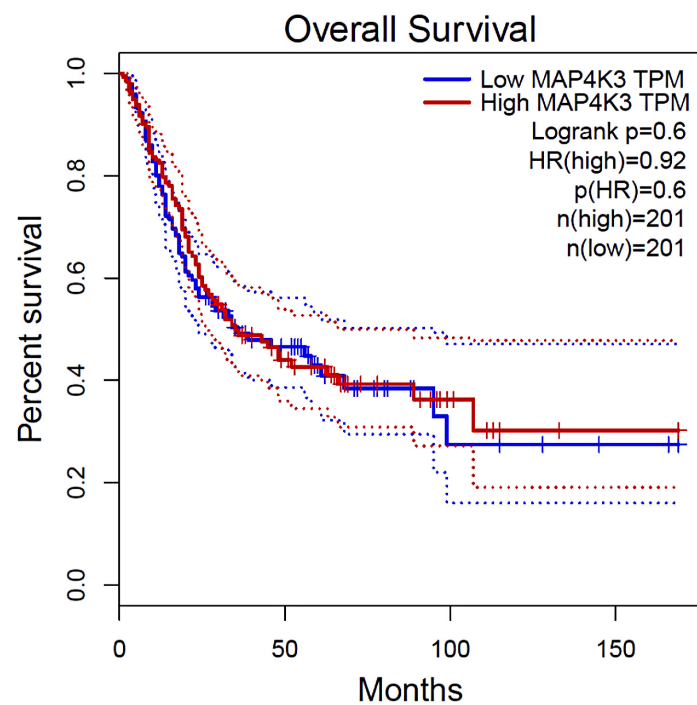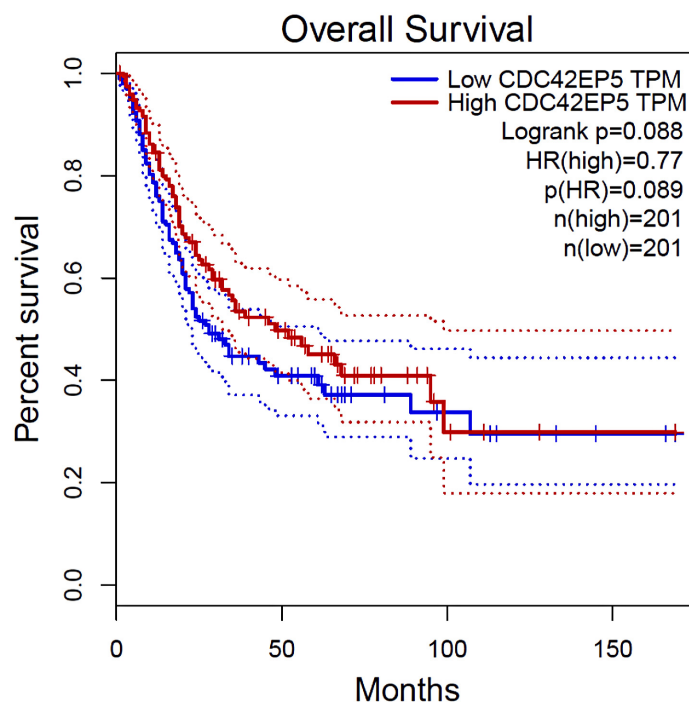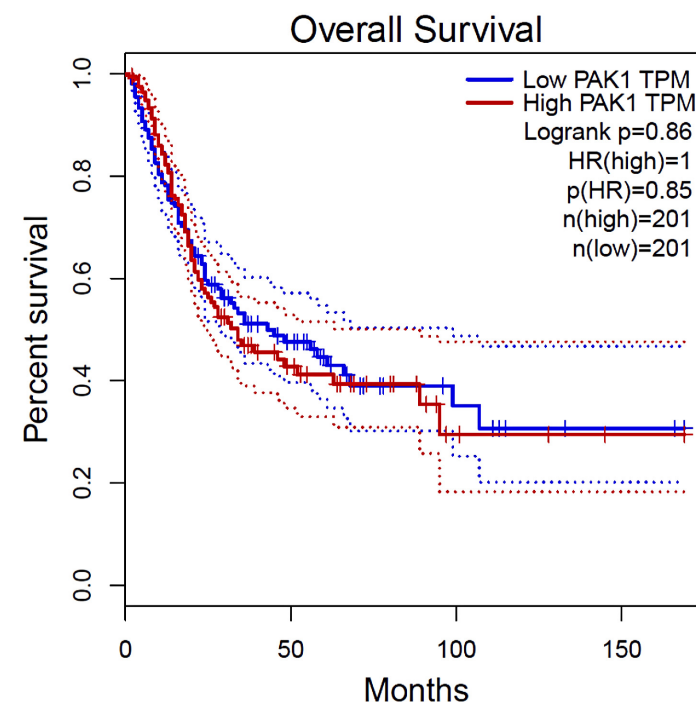

Supplement: Supplementary file 1 [file biomedicines-09-01054-s001.zip › Figure S1.pdf]

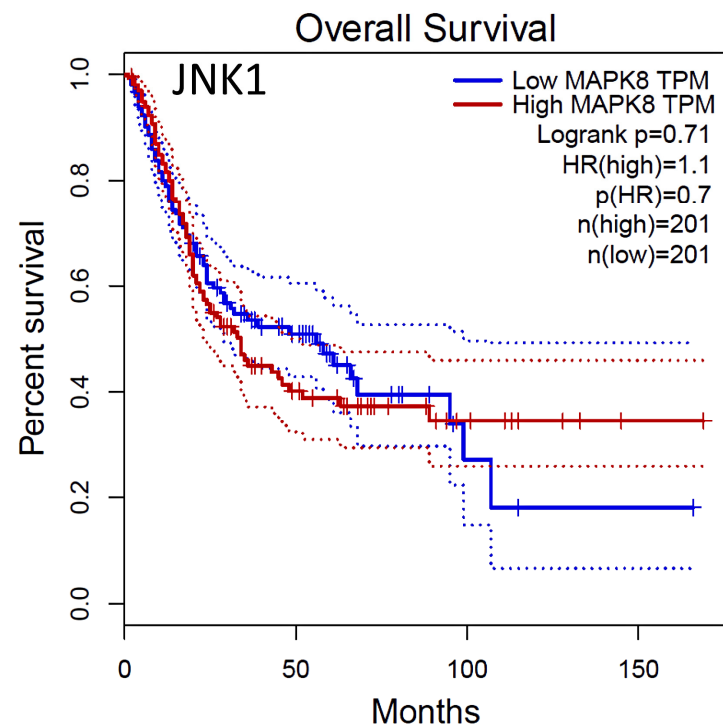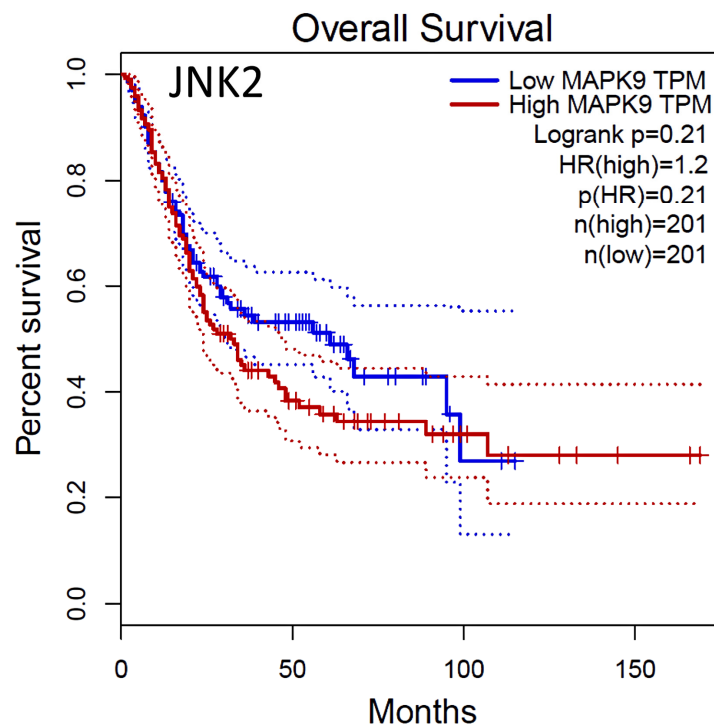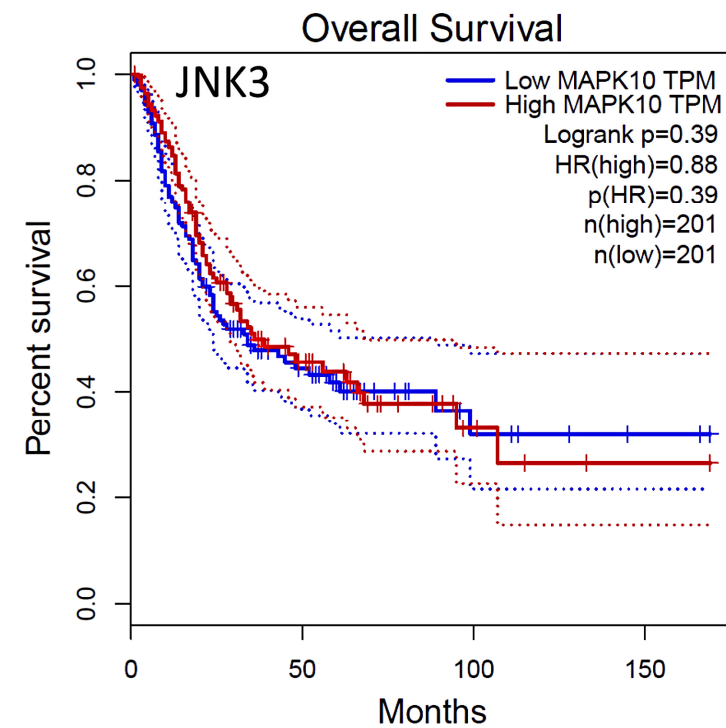

Supplement: Supplementary file 1 [file biomedicines-09-01054-s001.zip › Figure S2.pdf]
